# Lipid-based oral formulation in capsules to improve the delivery of poorly water-soluble drugs

**DOI:** 10.3389/fddev.2023.1232012

**Published:** 2023-08-24

**Authors:** Popat Mohite, Sudarshan Singh, Anil Pawar, Adinath Sangale, Bhupendra G. Prajapati

**Affiliations:** ^1^ A.E.T.s St. John Institute of Pharmacy and Research, Palghar, Maharashtra, India; ^2^ Department of Pharmaceutical Sciences, Faculty of Pharmacy, Chiang Mai University, Chiang Mai, Thailand; ^3^ M.E.S.’s College of Pharmacy, Ahmednagar, Maharashtra, India; ^4^ Shree S. K. Patel College of Pharmaceutical Education and Research, Ganpat University, Kherva, Gujarat, India

**Keywords:** oral drug delivery, lipids, BCS-II, bioavailability, improved solubility, stability

## Abstract

Poorly water-soluble drugs demonstrate significant challenge in pharmaceutical development, which is linked to their limited oral bioavailability and therapeutic efficacy. To overcome these limitations, lipid-based formulations have emerged as a promising approach to enhance the delivery of such drugs. Moreover, encapsulation within capsules to provide a convenient dosage form for oral administration. The encapsulation techniques are optimized to ensure uniform drug content and efficient encapsulation efficiency. Several investigations demonstrated that the lipid-based formulations in capsules significantly improved the solubility and dissolution rate of poorly water-soluble drugs compared to non-lipid formulations. Additionally, the encapsulation of lipid-based formulations protected the drug against degradation and improved its stability. Overall, incorporating lipid-based formulations in capsules represents a promising strategy for enhancing the delivery of poorly water-soluble drugs with improvement in solubility, dissolution, stability, and bioavailability, overcoming the challenges associated with these challenging drug molecules. The review focussed a brief on utilization of lipids in capsule form to improve therapeutic efficacy of poorly soluble, dissolution and bioavailability of drugs.

## Introduction

Oral delivery is the most widely used mode of administration because it circumvents intravenous side effects such as medication or blood release, catheter infection, and thrombosis (Roger et al., 2011). Oral administration is constrained by decreased oral bioavailability due to problems with the drug’s physicochemical properties, including poor solubility, low permeability, instability, and rapid metabolism (Prabhu et al., 2005; Alqahtani et al., 2021).

Numerous molecules with potential therapeutic properties have been developed because of advances in drug design. However, the bulk of recently discovered chemical entities have high molecular weights, are categorised as biopharmaceutical classification system (BCS-II), have high membrane permeabilities, and have low water solubilities. Because of these two conditions, oral medicine bioavailability is decreased (Amidon et al., 1995). Low dissolution and restricted absorption result from these drug’s restricted solubilities. This poor solubility leads to significant inter- and intra-subject variability, limited oral bioavailability, and a lack of dosage proportionality (Porter and Charman, 2001). Additionally, when taken with food, some of these drugs, like halofantrine (Charman et al., 1993) and danazol, have a higher bioavailability (Humberstone et al., 1996). To create these treatments in a safe and efficient way, a balance between bioavailability, toxicity, and disposition inside the body must be maintained. There have been reports of methods to address different solubility and permeability issues, including surfactants, solid dispersions, micronization, complexation with cyclodextrins, and solid dispersions (Aungst, 1993; Humberstone et al., 1996).

Recently, lipids have received a lot of attention as delivery methods for drugs with limited water solubility (Pouton, 2006). The availability of novel lipid excipients with acceptable regulatory and safety profiles, along with their ability to boost oral bioavailability, has made it possible to develop lipid-based formulations as a mechanism of drug administration. Lipid-based drug delivery (LBDD) systems have gained a lot of attention in recent years because of their ability to improve the solubility and bioavailability of drugs with low water solubility (Jannin et al., 2008). How successfully a medication is absorbed from a lipid-based formulation depends on several factors, including particle size, emulsification, the speed of dispersion, and the precipitation of the drug upon dispersion (Pouton, 2000). Examples of lipid-based formulations include oil solutions or suspensions, emulsions, and self-micro or self-nano emulsifying drug delivery systems (SMEDDS/SNEDDS) (Hauss et al., 1998; Pouton, 2000). Commercially available medications with lipid-based formulations include efavirenz (Sustivas), saquinavir (Fortovases), ritonavir (Nor-virs), and clofazimine (Lamprenes). With the proper lipid vehicles, formulation strategies, and careful system design, lipid-based drug delivery systems can be effective (Dahan and Hoffman, 2008).

A water-insoluble drug can be formulated as a lipid-based formulation when the drug itself is an oil-like substance (such as ethyl icosapentate, tocopherol nicotinate, teprenone, indomethacin farnesil, and dronabinol), or when conventional formulation techniques like granulation or soluble liquids in capsules do not increase the oral bioavailability (Hauss, 2007). Different lipid-based systems can be created depending on the excipients and formulation variables, ranging from straightforward oil solutions to intricate mixtures of oils, co-solvents, surfactants, and co-surfactants (Pouton and Porter, 2008). These systems can, in fact, be converted by several techniques into solid intermediates (powders, granules, and pellets), which can then be placed in hard gelatine capsules or compacted into tablets after being combined with suitable tableting excipients (Jannin et al., 2008).

### General routes of lipid-based drug delivery system

Because it is inexpensive, painless, and less likely to result in adverse effects, including injection-site responses, oral administration is the most common. Other methods, such as parenteral, ocular, intranasal, and vaginal routes, are also used to give lipid-based drug delivery system **(**LBDDS) (Nakmode et al., 2022a). The topical or dermal/transdermal routes of lipid-based drug administration allow for the easy penetration of the skin layer by nanosized lipid particles (Akombaetwa et al., 2023). As a result, the route of administration of the lipid formulation can be changed to suit patient needs.

### Lipid-based drug delivery system via oral route

Medicines that are weakly water soluble, such as BCS classes II and IV medicines, have been administered orally using LBDDS (Shrestha et al., 2014a). LBDDS are a desirable option for oral delivery because of their inherent biocompatibility, versatility in particle size, ease of scale up, and cost. Oral bioavailability is increased by lipid-based medication delivery, which reduces enzymatic and chemical drug breakdown. Another essential component of lipid-based formulation is their high surface area, which allows them to demonstrate strong resistance to enzyme attack by intestinal lipases and shields the medicine from the unfavourable surroundings of the gastric system (Li et al., 2012).

It has recently been shown that oils increases the absorption of lipid-soluble vitamins, including lipid-soluble vitamins A, D, E, and K. This practical illustration from daily life demonstrates how the composition of lipids can aid in the absorption of active substances. In a recent study, diabetic rats were used to examine the oral bioavailability of solid lipid nanoparticles (SLNs) carrying insulin. This lipid-based drug delivery strategy kept the insulin from entering the gastrointestinal environment, and it also had a five-fold better bioavailability than the insulin solution (Chime and Onyishi, 2013; Ansari et al., 2016).

**FIGURE 1:**
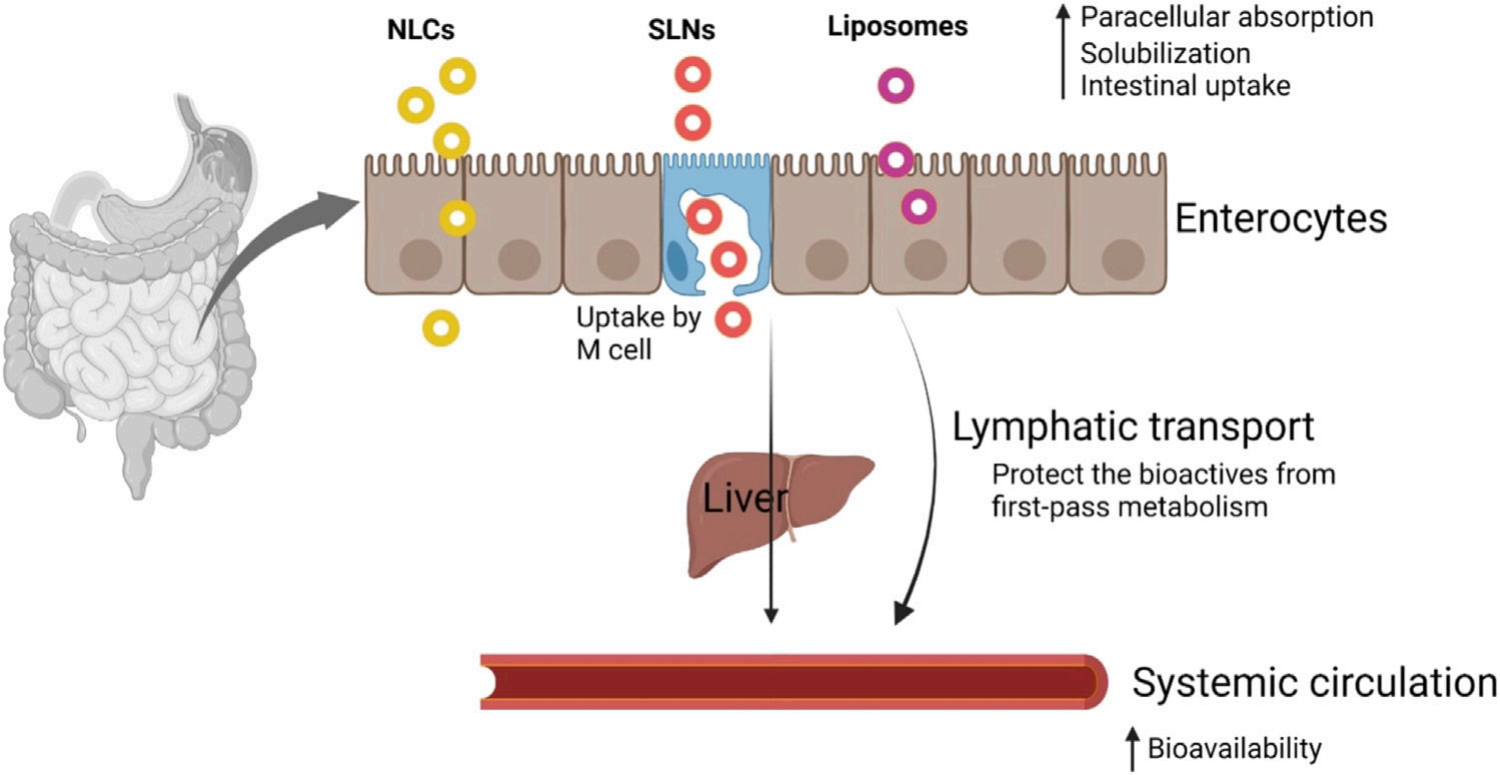
Transport mechanism of nanosized lipid-based delivery systems. Reproduced with permission from Subramanian (2021).

In a different study, SLNs of amphotericin B were employed to create an efficient oral delivery route. When kept in the refrigerator, the formulations were discovered stable up to 3 months. Additionally, they were found to have better efficacy than the commercially available formulation (Fungizone), little renal toxicity, and stability across a range of gastrointestinal settings. Despite their many advantages, lipid-based formulations are overcoming several obstacles. Additionally, LBDDS are still undergoing clinical trials to demonstrate their oral efficacy (Bianco et al., 2010). A generalized transport mechanism of orally administered nano-sized lipid-based delivery systems is presented in Figure 1 (Subramanian, 2021).

### Lipid-based drug delivery system via parenteral route

Many medications, including peptides and proteins, cannot be taken orally because of an enzymatic breakdown in the GIT. These medications can be administered repeatedly by parenteral infusion. SLNs is a promising drug delivery system for long-term, controlled-release parenteral administration, are a lipid-based formulation. SLNs can easily circulate in the microvascular system when given intravenously because of their nano size and display their therapeutic effects (Wissing et al., 2004).

### Lipid-based drug delivery system via topical route

Lipid-based products are used to deliver drugs when applied topically because they have different favourable properties, such as small particle size, which makes it easier for medications to penetrate skin and ensure intimate contact with the stratum corneum (Pardeike et al., 2009). Due to their lipid composition that is non-irritating and non-toxic, alongside their ability to release medication for extended periods while limiting systemic drug absorption, they are suitable for topical therapy on damaged or inflamed skin (Wissing and Müller, 2003).

### Lipid-based drug delivery system via ocular drug delivery

LBDDS is used to cure the different severe optical conditions, such as glaucoma, infections, ocular irritation, and diseases that impact the structures of the posterior eye. Therefore, several anti-infectious, anti-inflammatory, and anti-glaucoma medications are supplied as eye drop using such delivery systems (Farid et al., 2017).

### Lipid-based drug delivery system via pulmonary route

Studies have recently turned their focus to the pulmonary route as a non-invasive alternative for both local and systemic drug delivery because of the lung’s large surface area for drug absorption and rapid drug absorption because of the alveolar epithelium’s ultra-thinness, high vascularization, and absence of first-pass metabolism (Li et al., 2010). The disclosed findings suggest SLNs may be very beneficial for both local therapy and systemic drug administration for severe airway disorders. To distribute medication into the lungs, dry powder products or aqueous solutions of lipid nanoparticles are used (Hauss, 2007).

### Mechanism of drug release from the LBDDS in oral route

The release of drug from a lipid-based product depends on two primary principles: digestion and absorption.

#### Digestion

When lipid-based products are taken orally, lingual and stomach lipases crash the triglyceride of the lipid-based product and exogenous dietary fat. The propulsion, grinding, and retropulsion actions of the stomach mechanically combine the lipid product and food into the GI content. After that, it combines aqueous stomach fluid with fat digestion byproducts to create a crude emulsion-like substance. Pancreatic lipase and its cofactor co-lipase 203 mainly work at the sn-1 and sn-3 sites of triglycerides to create monoglycerides and free fatty acids, which are then broken down into diglycerides, monoglycerides, and fatty acids later in the small intestine. In order to create lysophosphatidic choline and fatty acids, pancreatic phospholipase hydrolyzes phospholipids at the sn-2 location.

Endogenous biliary lipids, such as bile salt, phospholipids, and cholesterol, are released from the gallbladder because of exogenous lipids in the small intestine. By-products of lipid digestion such as monoglycerides, fatty acids, and Lys phospholipids are combined with bile salts to create a range of colloidal forms, including as micelles and unilamellar and multilamellar vesicles. The small intestine’s capacity to penetrate and absorb substances that require lipid digestion is increased by these generated lipid metabolites (Ros, 2000; Phan and Tso, 2001).

#### Absorption

Drug absorption can be influenced by lipid-containing formulations in several ways. They can stimulate the lymphatic transport of medicine by affecting the gut environment (Gershkovich and Hoffman, 2007; Fouad et al., 2011). But it was found that when API was dissolved in a LBDDS, the medication’s absorption was superior to that of traditional solid dosage forms (Umeyor et al., 2012). Its capacity too quickly wet water insoluble drug particles in the lipid matrix is one of its characteristics that promotes absorption. This could be the outcome of the formulation and the addition of a surfactant. The likelihood of the medication becoming trapped inside micelles is also increased by the lipid matrix (Joshi and Shah, 2005). The rate-limiting stage for the absorption of water insoluble drug is dissolution. This lipid’s job is to enhance dissolution by combining various colloidal particles with bile components, which eventually enables them to use micellar solubilization to keep a sizable amount of lipophilic medication in solution (Porter et al., 2007)., the procedures for lipid-based medication formulations start with dispersion to form lipid or emulsion droplets, and bile acids and lipolysis solubilize the byproducts of digestion to create colloidal mixed micelles. The drug is expected to be absorbed by the mucosal cells of the gut wall once it has separated from the bile salt and emulsion oil droplets (Pouton, 2000; Cannon et al., 2018).

### Lipid-based formulation classification system

A functioning model for the Lipid Formulation Classification System (LFCS) was presented in 2000 (Pouton, 2006). After that, a brand-new formulation type was included in 2006 (Pouton, 2000). The primary aim of the LFCS is to simplify *in vivo* investigations in order to facilitate the detection of the optimum products for precise medications based on their physicochemical property (Pouton and Porter, 2008). In recent years, the pharmaceutical industry has conducted more open research on the LFCS to reach an outline for evaluating the effectiveness of lipid-based products. Table 1 displays the various LFCS classes. Most goods available are Type III systems, which are varied and comprise a range of ingredients that are both oil- and water-soluble. Consequently, depending on the proportion of oils to water-soluble chemicals, this group has been further separated into Type III A (oils) and Type III B (water-soluble) (Kalepu et al., 2013a).

**TABLE 1:** Various lipid formulation classification system classes with their excipients, features, and advantages (Pouton and Porter, 2008; Kalepu et al., 2013b).

Formulation type	Excipients	Features	Advantages	Disadvantages
Type I	Oils (surfactants are unnecessary)	Requires digestion; non-dispersing	great compatibility with capsules	the formulation requires a highly lipophilic drug because solvent capacity is poor
Type II	Required Water-insoluble surfactants and oils	Without water-soluble components, SEDDS are formed	Probability of holding solvent capacity after dispersion	Turbid oil-in-water dispersion with particle size ranging from 0.25 to 2 μm
Type III A (fine emulsion)	Oils, surfactants, and co-solvents (both water-insoluble and water-soluble excipients)	Water-soluble components are used to produce SEDDS and SMEDDS.	Without digestion, drugs are absorbed	chances of losing solvent capacity
Type III B (micro emulsion)	Oils, surfactants, and co-solvents (both water-insoluble and water-soluble excipients can be required)	SEDDS/SMEDDS are produced using low-oil quantity and water-soluble excipients	clear dispersion and Without digestion, drugs are absorbed	chances of losing solvent capacity
Type IV	Water-soluble surfactants and co-solvents (no oils required for formulation)	the micellar solution formed by dispersing formulation	suitable for many drugs as a solvent	chances of losing solvent capacity on dispersion may not be easily absorbed or difficulties in digestion

## Candidate drugs for lipid-based formulation

In the formulation of LBDDS, compound with low water solubility, specifically, those in BCS class II and IV, are frequently considered. Different physical and chemical factors might lead compounds to fall between BCS II and IV; as a result, figuring out the reason for the low solubility could be important. Because of their rigid crystal structure, poorly water-soluble compounds are known as “brick dust” and cannot be synthesised as LBDDS. Another class of compounds, known as “grease-balls,” has a high lipophilicity (log P) and significantly lower melting temperatures. Between these two simplified classifications of poorly soluble compounds, there is undoubtedly a spectrum, with most therapeutic molecules sitting somewhere in the middle. If a chemical has grease-ball characteristics and standard formulation techniques do not produce the desired bioavailability, it may be advantageous to increase solubility by adding surfactants and/or lipid-based excipients. The solubility of a substance, if it is a “brick-dust” molecule, is often constrained in lipids, but it may be considerably boosted in surfactants and co-solvents. Because of this, not all substances with poor water solubility and/or an elevated log P will have good solubility for excipients utilised in LBDDS (Mu et al., 2013).

A sound and organised method for establishing the product space for LBDDS is to use design of experiments (DoE). Drug solubility, aqueous dispersion, and colloid structures are common output features. When solubility is considered an output in the DoE, formulation mixes may achieve higher solubility than individual excipients (Holm et al., 2006).

### Selection of additives for lipid-based formulation

Lipid additives are obtainable from a range of additive providers. Because these lipids influence the process of absorption, it is crucial to understand the characteristics of different excipients (Pouton and Porter, 2008).

The main variables affecting the selection of additives for products based on lipids such as solubility of lipid excipients, dispersion, digestion of lipid and absorption. Other factors include Safety consideration like toxicity irritancy, Solvent capacity of lipid-based formulation system, Excipients Miscibility, Excipients digestibility, Chemical stability and purity of lipid excipients, Compatibility of lipid excipients with capsule material, Dispersibility of excipients and their role in facilitating self-dispersion of the formulation, Morphology (melting point) at room temperature and the cost of the raw material, etc.

#### Solubility

Although lipids make up most of the products, additional surfactants may also be needed as a hydrophilic co-solvent to increase solubilization and dispersion capabilities. Most of the lipids used in these products have a predetermined “required HLB” value, which is often obtainable from vendors and correlates to the ideal HLB for the surfactant combination needed to emulsify the oil in water. The hydrophilic-lipophilic balance (HLB) number is used to categorise surfactants, with a lower value (10) indicating more lipophilicity and a larger value (10) indicating strong hydrophobicity (Constantinides, 1995; Strickley, 2004; Kotta et al., 2018).

#### Dispersion

Formulations with suitable drug candidate solubility should undergo aqueous phase emulsification and dispersion testing. A preliminary screening can be done by using a microscope to look at the formulation after it has been combined with water. At the water/formulation interface, a successful emulsification is distinguished by vigorous mixing, as well as diffusion and stranding mechanisms. It is useful to measure the particle size of emulsion droplets using the laser light scattering method in order to select efficient formulations. A common method for identifying the kinds of structures that result from emulsification and characterising how a product behaves along a dilution path is the production of ternary phase diagrams (Cannon and Long, 2018).

#### Digestion of lipid

Intestinal lipases have a substantial impact on how lipid-based formulations behave in the GI tract, hence this should be measured when developing such formulations. Long known for their ability to break down non-dispersible but digestible lipids like triglycerides into mono- and diglycerides and fatty acids, this will emulsify any leftover oil. Lipases can do this. Large quantities of surfactants might not be required to produce the small particle sizes and large surface surfaces for release of the drug. To enhance dispersion, Poulton projected a categorization scheme for lipid-based products depends on excipient and digestion (Pouton and Porter, 2008).

#### Absorption

Any oral lipid-based formulation’s principal aim is to successfully distribute the medication to the intestinal mucosal cells. Small intestine’s play a significant role in medication absorption and lipid digestion (Shrestha et al., 2014b). Before the digestive contents are dissolved by bile acids and lipolyzed to make colloidal mixed micelles, the ingredients are first circulated to produce lipid droplets (for type I formulations) or emulsion droplets (four types II–III formulations). In order for the medication to be uptake by the mucosal cells of the intestinal wall, it is therefore expected that it would split from the bile salt and emulsion oil droplets (Chen et al., 2012; Cannon and Long, 2018).

#### Lipid additives used in the pharmaceutical product development

The applications of the lipid additives, which have capable potential to create lipid products, are discussed below and summarized in Table 2.

**TABLE 2:** Lipid excipients used in the lipid-based formulations (Gunstone and Harwood, 2007; Santra and Majee, 2022).

Water insoluble lipid excipients	Triglycerides		Surfactants	Co-solvents	Additives
	Medium chain	Long chain			
Bees wax, oleic acid, soy fatty acids, corn oil mono-di-triglycerides, medium chain (C8/C10) mono-and diglycerides, propylene glycol esters of fatty acids	Caprylic/capric triglycerides derived from coconut oil or palm seed oil	Hydrogenated soyabean oil, Hydrogenated vegetable oil, Corn oil, Olive oil, Sesame oil	Polysorbate 20 (tween 20), Polysorbate 80 (tween 80), sorbitan mono laurate (Span 20, D-α -tocopherol PEG 1000 succinate (TPGS), Polyoxyl 40 hydrogenated castor oil (cremophor RH40), PEG 300 oleic glycerides (Labrafil sM-1944CS)	Ethanol, glycerol, propylene glycol, and polyethylene glycols (PEG)-400	-tocopherol, -carotene, propyl gallate, butylated hydroxyl toluene (BHT), or butylated hydroxyanisole (BHA) used as antioxidants

#### Glyceryl dibehenate

Glyceryl dibehenate is produced when behenic acid and glycerol are esterified. The creation of lipid-based nanocarriers such SLNs, nanostructured lipid carriers (NLCs), and nanoparticles is frequently done using this method (Nakmode et al., 2022b). By combining a changed high shear homogenization technique with an ultra-sonication procedure, Abdelbary and Fahmy could create SLNs with various glyceryl dibehenate and glyceryl stearate concentrations. Glyceryl dibehenate and glyceryl stearate were included as lipid components. Glyceryl dibehenate produced larger size SLNs with higher entrapment and noteworthy prolonged release of diazepam when compared to formulations containing glyceryl stearate (Abdelbary and Fahmy, 2009). Moreover, the use of glyceryl dibehenate in the production of SLN for topical application was studied by Mancini and others. Utilising glyceryl dibehenate as a solid lipid and polyoxyethylene sorbitan monooleate as a surfactant, SLNs were produced utilising the fusion emulsification method. Etofenamate and ibuprofen medication molecules are inserted into a hydrogel made of 2% hydroxy propyl methylcellulose gel through the gelation of the SLN solution. Lipid nanoparticle encapsulation of the medicine minimises drug leakage while simultaneously shielding it from the environment. The formulations were discovered to have a 90% encapsulating efficiency and suitable particle size (250 nm). Additionally, SLN-containing hydrogel showed improved drug penetration compared to previous hydrogel formulations (Mancini et al., 2021).

##### Dynasan

Glyceryl trimyristate (Dynasan^®^114), glyceryl tristearate (Dynasan^®^118), and glyceryl tripalmitin (Dynasan^®^116), among others, are saturated, even numbered, and unbranched single fatty acids derived from plants. Dynasan^®^ is also used as an excipient to control the pace of dissolution, depending on the concentration. In various grades, including 114, 116, and 118, Suvarna and others created SLNs in collaboration with Dyanasan (Olbrich et al., 2002b). The processes of hot homogenization and ultra-sonication were employed to produce solid lipid nanoparticles. Three lipids and 2 different concentrations were employed to prepare each of the six SLNs formulations. Particle size, zeta potential, and the PDI index (poly dispersity index) were employed to describe SLNs. The formulation of the rosuvastatin SLNs improved oral bioavailability by 2.2 times when compared to a control suspension because it avoided the effects of first pass metabolism by exploiting the lymphatic transport channel (Dudhipala and Ay, 2020).

##### Mixed glycerides and polar oils

Mixed glycerides are created by partially hydrolyzing vegetable oils. Depending on the initial substance (triglyceride) and the level of hydrolysis, mixed glycerides have different compositions. Medium chain mixed glycerides help with emulsification, have a superior solvent capacity, and are less likely to oxidise. The solvent capacity and dispersibility of the product are increased by these polar oily excipients (Suvarna et al., 2015). Fenofibrate was planned in representative Type II, IIIA, IIIB and IV self-emulsifying/micro emulsifying lipid delivery systems (SEDDS and SMEDDS designed for oral administration) using various medium-chain glyceride components, non-ionic surfactants and co-solvents as excipients (Mohsin, 2012).

#### Glyceryl distearate

The primary ingredients in glyceryl distearate are stearic acid and palmitic acid esters. Due to its potent anti-friction properties, it is perfect for both capsule filling and taste muffling (Strickley, 2007). The principal uses of glyceryl distearate involve melting it into granules and encapsulating it in a matrix. Forster and others studied twin-screw melt granulation as a method to create taste-masked ibuprofen granules using glyceryl distearate as a lipid binder (Forster and Lebo, 2021). It was found that the release of ibuprofen from the granules was slower than that of pure API and physical mix. By constructing a physical barrier, such as matrix encapsulation, in which the medication is encased in a lipid matrix and only a tiny amount is left on the surface, which is tolerated when it dissolves into the saliva, glyceryl distearate inhibits dissolution (Becker et al., 2015).

#### Glyceryl caprylate

By fractionating vegetable oil fatty acids and certain oils, fats, and fat acids used in lipid synthesis, these mono- and di-glycerides emulsifiers are created. Kim and others advise employing a micro-emulsion formulation to increase Rebamipide’s oral bioavailability. A medium-chain glyceride (glyceryl caprylate), ethanol as a co-surfactant, and polyoxyethylene esters of 12-hydroxy stearic acid serve as the surfactants in the lipid phase. The micro-emulsion increased Rebamipide’s oral bioavailability and dissolving profile, showing no signs of intestinal toxicity (Kim et al., 2017).

#### Glyceryl stearate

In o/w emulsions, glyceryl stearate, which has a monoester content of 40%–55%, serves as a co-emulsifier and consistency enhancer. To keep them out of the eating environment and maintain their effect *in vivo* (slow release), Prombutara and others developed SLNs containing nisin. *Listeria monocytogenes* DMST 2871 and *Lactobacillus* plantarum TISTR 850 were resistant to SLNs loaded with nisin for up to 20 and 15 days, respectively, as opposed to 1 and 3 days in the absence of nisin (Prombutara et al., 2012).

#### Caprylocaproyl macrogol-8 glyceride

Caprylocaproyl macrogol-8 glyceride, a non-ionic oil-in-water surfactant, is soluble in both oil and water. It has a wide range of applications in topical and transdermal formulation due to its emulsifying and absorption-enhancing capabilities (Zhou et al., 2015; Chopra et al., 2022). Zhou and associates discovered that the co-emulsifier caprylocaproyl macrogol-8 glyceride (labrasol) increased the oral bioavailability of resveratrol. The suppression of intestinal glucuronidation handled this improvement (Zupančič et al., 2016).

#### Lauryl Polyoxyl-32 glycerides

In lipid-based formulations, lauryl polyoxyl-32 glycerides are frequently employed as a non-ionic surfactant. Mono-, di-, and triglyceride fatty acid PEG esters are a group of transportation agents known as Lauroyl Polyoxyl-32 glycerides. Lauroyl polyoxyl-32 glycerides can emulsify to a fine dispersion or cause micro-emulsion when in contact with an aqueous media. The surfactant may dissolve in fluids and is non-ionic. Owing to its surface-active quality, it promotes an increase in API’s solubility and wetting capacity both *in vitro* and *in vivo*. It functions as a coherence agent (thickener) in topical formulations (Panigrahi et al., 2018).

#### Medium-chain triglycerides

They offer improved skin absorption and skin spreadability. Caprylic/Capric Triglyceride functions as a medication carrier, solvent, emollient, and penetration enhancer in oral and topical preparations. The self-emulsified drug delivery system also contained capric triglyceride, polyoxyethylated castor oil, diethylene glycol mono ethyl ether, and CsA. Three distinct coating thicknesses low, medium, and high of ethyl cellulose and pectin polymers applied to Sm Pill mini spheres. The bioavailability of CsA made from SmPill mini spheres was compared with commercial Neoral^®^ PO and Sandimmun^®^ iv using a pig model (Keohane et al., 2016).

#### Water-insoluble surfactants

There is a class of lipid additives with an intermediate hydrophilic lipophilic balance (HLB of 8–12) that absorbs at oil–water interfaces. Depending on the quantity of ethoxylation, they are only soluble in a certain amount of water. They can create emulsions when shared and are occasionally referred to as “dispersible” in water. Although these substances could form micelles, they lacked the hydrophilicity needed to self-emulsify. Oleate esters, such as polyoxyethylene (20) Sorbian trioleate (Tween-85) and polyoxyethylene (20) glyceryl trioleate (TagotTO), often have HLB values between 11 and 11.5. Tween-85 functions similarly, however, a blend of Tween80 and Span-80 with an average HLB value of 11 (Wakerly et al., 1986; Pouton, 1997).

#### Water-soluble surfactants

The creation of SEDDS most usually employs these surfactants. By dissolving in pure water above their critical micellar concentration in delivery systems, materials with an HLB value of 12 or above can form micellar solutions at low concentrations (Ivanov, 2023). These chemicals can be created by mixing hydrolyzed vegetable oils and polyethylene glycols (PEG). Alkyl ether ethoxylate, a common surfactant (such as cetostearyl alcohol ethoxylate, commonly known as “cetomacrogol”), is a product of the reaction of alcohols with ethyleneoxide. When sorbitan esters and ethylene oxide (mostly ether ethoxylates) mix, polysorbates are created (Rowe et al., 2009). Examples include the ethoxylated hydrogenated castor oils (Cremophor RH40 and RH60), which are made by hydrogenating vegetable oils. Another well-liked non-hydrogenated oil is Cremophor EL (ethoxylated castor oil) (Cuiné et al., 2007; Grove et al., 2007). By blocking efflux pumps, cremophor improves absorption, albeit the mechanism of inhibition is unclear (Gibson, 2007).

##### Co-solvents

Co-solvents are used in most commercially available medicinal preparations to speed up the solubilization process (Strickley, 2004; 2007). Some of the frequently utilised Co-solvents include ethanol, glycerol, propylene glycol, and polyethylene glycols (PEG)-400. They help systems with a high concentration of water-soluble surfactants disperse more easily and improve the solvent capacity of medicinal formulations. These Co-solvents do, however, have significant practical constraints, such as the precipitation of the medication that has been solubilized from the solvent because of the solvent’s diminished capacity after dilution (Yalkowsky, 1999). Some Co-solvents and low molecular weight solvents are incompatible with oils and capsule shells, respectively (Cole et al., 2008).

##### Selection of excipients for lipid-based formulation

Lipid additives are obtainable from a range of additive providers. Because lipids influence the process of absorption, it is crucial to understand the characteristics of different excipients (Jannin et al., 2008). The following are the main variables disturbing the selection of additives for formulations depends on lipids solubility, dispersion, digestion, and absorption.

##### Other additives

Tocopherol, carotene, propyl gallate, butylated hydroxyl toluene (BHT), and butylated hydroxyanisole (BHA) are a few lipid-soluble antioxidants that can be utilised to protect the preparation against oxidation (Padley, 1994; Kalepu et al., 2013b).

### Formulation approaches

Lipid-based products can be effectively designed if the formulation objectives are given enough thought. The systematic approach entails selecting an appropriate formulation method for the intended dosage form, designing appropriate animal models to estimate performance *in vivo*, and simultaneously optimising the drug product for solubility, stability, and compatibility. Pre-selection of additives is based on their melting point, fatty acid composition, HLB value, digestibility, and degradability.

#### Oily liquids

Only triacylglycerols, which are incredibly lipophilic and soluble in oils, can make certain medications, like steroids, soluble. To create an oily liquid, such medications must be dissolved in oil. However, the medicine’s use in oil formulations is constrained or limited because a sizable amount of oil is necessary to liquefy a single dose of the drug (Čerpnjak et al., 2013). According to a study conducted by Guruge and others, Type III lipid-based formulations (LBFs) are used to integrate poorly water-soluble drugs with oils, surfactants, and co-solvents in order to deliver the drugs into the systemic circulation. The drug’s solubility can be affected by the colloidal phases that are generated in the gastrointestinal tract when the formulation comes into contact with bile and other substances present in the GI tract. In this study, modeled the internal structures of five type III LBFs of loratadine containing poly (ethylene oxide) nonionic surfactants polysorbate 80 and polyoxyl hydrogenated castor oil (Kolliphor RH40) using long-timescale MD simulations (0.4–1.7 μs) (Guruge et al., 2021).

#### Mixed micelles

Several molecular species make up these systems. These micelles resemble lipid bilayers and have a disc-like form. The detergent molecules serve as a water barrier around the borders of the lipid molecules in detergent-lipid mixed micelles. Methotrexate has boosted anti-tumor activity against malignancies that are drug-resistant when coupled with polymeric mixed micelles (made of Pluronic F127 and P105) (Shrestha et al., 2014a). The investigation conducted on the formulation and topical efficacy of novel lipid-based preparations of diclofenac aqueous gel containing mixed micelles, diclofenac lotion, and diclofenac lipogel containing egg lecithin, isopropyl myristate, propylene glycol, and ethanol were developed using Carbomer 934. The study findings indicate that diclofenac lotion and lipogel may be more appropriate formulations when compared to conventional topical dosage forms (Parsaee et al., 2002).

#### Self-emulsifying systems

These systems can emulsify because, with oily phase, they also contain one or more surfactants. The lipophilic medication dissolves in oil. When the surfactant help disperse the oily vehicle in the GI fluid, a micro-emulsion is created. Depending on the size of the emulsion particles, these systems can also be categorised as SMEDDS or SNEDDS. The medication, oily carriers, a surfactant, a co-surfactant, and co-solvents are typically included in the product (Kumar et al., 2010). Clobetasol propionate-loaded micro-emulsion based gel prepared by Patel et al. showed that higher drug permeation into the skin micro-emulsion (60.33% ± 4.67%) compared with the marketed product (37.77% ± 0.77%) with better retention in the skin and minimal irritation potential thus proved to be a promising formulation for the effective treatment of vitiligo (Patel et al., 2013).

#### Nanoemulsion

Although there are many other approaches, oral nano emulsions (NEs) have been suggested as a wonderful option, since they may eventually increase the solubility and bioavailability of lipophilic medications. At the moment, NEs are heavily preferred for the Class 2 and 4 classes. These nano-dispersion formulations have sizes between 20 and 500 nm, behave isotopically, and have unstable thermodynamic properties. They include a co-surfactant, a surfactant, and oil. It is very effective at encapsulating kinetically stable active compounds due to the extremely small droplet size. These brand-new, lipid-based drug delivery devices, which have diameters under 1 m, are the focus of extensive research (Tayeb and Sainsbury, 2018). Intranasal based Nano emulsion was prepared by Mahajan et al. where the optimized Nonemulation showed a high percentage of drug targeting efficiency (2,919.261 ± 5.68) and nose-to-brain drug direct transport percentage (96.574% ± 0.76) proving that this system is a good carrier for saquinavir mesylate to CNS through intranasal route (Mahajan et al., 2014).

##### Liposomes

The spherical bilayer structures of liposomes are like to cell membranes. The most common lipids employed are phospholipids, which are amphiphilic in nature and have a hydrophilic head and a hydrophobic tail (fatty acid). These phospholipids form spherical bilayer structures when hydrated in water, with their hydrophilic sections facing outward and their hydrophobic portions facing each other (inward) (Tayeb and Sainsbury, 2018). The advantage of these systems is that hydrophilic medications can be integrated inside the aqueous internal voids of the globules, whereas hydrophobic pharmaceuticals can be incorporated inside the inner fatty acid layers. Liposomes facilitate the intravenous administration of hydrophobic drugs with low water solubility, such as amphotericin B, by mitigating the toxicity associated with such drugs (Janknegt et al., 1992).

##### Lipid nanoparticles

Regarding the therapeutic goal, these specific nanoparticulate systems have outstanding characteristics. Using physiological lipids promotes controlled medication release and lessens GI absorption’s variable absorption profile (Blasi et al., 2007).

##### Solid lipid nanoparticles

The possibility of SLNs to increase bioavailability and enable regulated, site-specific medication delivery has recently garnered a lot of interest. Therefore, they are likely candidates for oral intestinal lymphatic delivery carriers. SLNs are typically spherical particles with a solid lipid core matrix that can solubilize lipophilic substances (stabilised by surfactants). They come in sizes between 10 and 1,000 nm (Mukherjee et al., 2009). These lipid-based nanocarriers have become yet another ground-breaking and impressive technique for enhancing the bioavailability of oral medications. Using this method, it is possible to develop therapeutic formulations for medicines with poor solubility, such as those in BCS classes 2 and 4 [30]. NLC is used in place of SLN to address issues including drug ejection during storage and poor drug payload. Lipospheres, SLM, SLN, and NLCs are additional classifications for the lipid-based particulate system (Plaza-Oliver et al., 2021b). The influence of various process parameters, such as emulsification time, stirring rate and cooling condition on the particle size and zeta potential, was investigated by Olbrich et al. In this study, lipids such as trimyristin, tripalmitin, and a mixture of mono, di-and triglycerides were used (Olbrich et al., 2002a).

#### Lipospheres

Owing to their homogeneous gastrointestinal tract distribution and uniform drug absorption, multiple-unit drug delivery methods, such as nanoparticles, microparticles, micro-emulsions, and liposomes, offer an advantage over single-unit systems. The distribution strategy of these particle systems has toxicity issues because of the presence of organic solvent residues. Lipid microspheres, also known as lipo spheres, have been suggested as a unique fat-based encapsulating technology for medication delivery of bioactive compounds to address these problems (He, 2020). The solid hydrophobic lipid core that contains the active pharmacological component is disseminated or dissolved in a solid fat matrix that is stabilised by an outer layer of phospholipid molecules. Lipospheres are solid lipid-based particles with sizes ranging from 0.01 to 100. The advantages of lipospheres in extending plasma medication levels include enhanced bioavailability, a longer shelf life, and the prevention of drug hydrolysis. Attama and others conducted a study where they developed and assessed ceftriaxone sodium lipospheres dispersions for oral administration *in vitro*. Through the melt-emulsification method, Ceftriaxone sodium lipospheres were produced with the lipid matrix comprising 30%w/w Phospholipon^®^ 90H in Softisan^®^ 154 and varying quantities of PEG 4000. The adequate management of Ceftriaxone stability in oral formulation can be achieved through the tactical engineering of lipid drug delivery systems like lipospheres (Attama et al., 2009).

#### Solid lipid microparticles

The hydrophobic core of solid lipid microparticles, which are solid at body and room temperatures, is stabilised by an embedded layer of surfactant. In the stable lipid matrix, the active component is disseminated or dissolved (He, 2020). Solid lipid microparticles are created by substituting a solid (at room temperature) lipid component for the liquid oil in traditional oil-in-water (o/w) emulsions. They offer good *in vivo* tolerability and excellent biodegradability because they are mostly constructed of physiologically acceptable and biodegradable components. Because of their distinct and ideal size, the solid lipid microparts are precisely supplied by a variety of methods, including cutaneous, nasal, and pulmonary distribution.

#### Nanostructured lipid carriers

The primary structural components of the drug delivery systems known as nanostructured lipid carriers (NLCs) are solid and liquid lipids. NLCs have proven to have many benefits over conventional carriers when used for drug administration, including enhanced permeability, higher bioavailability, fewer adverse effects, long-lasting half-life, and tissue-targeted delivery (Fang et al., 2013). The efficiency of SLNs as drug delivery systems for cutaneous, topical, parenteral, and oral routes has been scrutinized. However, Gainza et al. changed the synthesis and revealed that NLCs could also deliver heat-sensitive drugs (such as proteins) (Gainza et al., 2014). They used it for the promotion of faster and more effective healing and suggest their future application to treat chronic wounds.

### Selection of the drug compound for lipid-based formulation

For the formulation of LBDDS, drugs with low water solubility, specifically, those in BCS class II and IV, are frequently considered. Different physical and chemical factors might lead compounds to fall between BCS II and IV; as a result, figuring out the reason for the low solubility could be important. Due to their rigid crystal structure, poor water-soluble compounds are known as “brick dust” and cannot be synthesised like LBDDS. Another class of compounds, known as “grease-balls,” has a high log P (lipophilicity) with notably lesser melting temperatures. Between these two simplified classifications of low soluble drugs, there is undoubtedly a spectrum, with most therapeutic molecules sitting somewhere in the middle (Kuentz, 2012).

If a chemical has grease-ball characteristics and standard product techniques do not produce the desired bioavailability, it may be advantageous to increase solubility by adding surfactants and/or lipid-based excipients. The solubility of a substance, if it is a “brick-dust” molecule, is often constrained in lipids, but it may be considerably boosted in co-solvents and surfactants. Because of this, not all substances with low water solubility and/or an elevated log P have good solubility for additives utilised in LBDDS (Kuentz, 2012).

A sound and organised method for establishing the product space of LBDDS is to use design of experiments. Drug solubility, aqueous dispersion, and colloid structures are common output features. When solubility is considered an output in the DoE, formulation mixes may achieve higher solubility than individual excipients (Kuentz, 2012).

### Recent advancement of lipid nanocarrier in oral drug delivery

**FIGURE 2:**
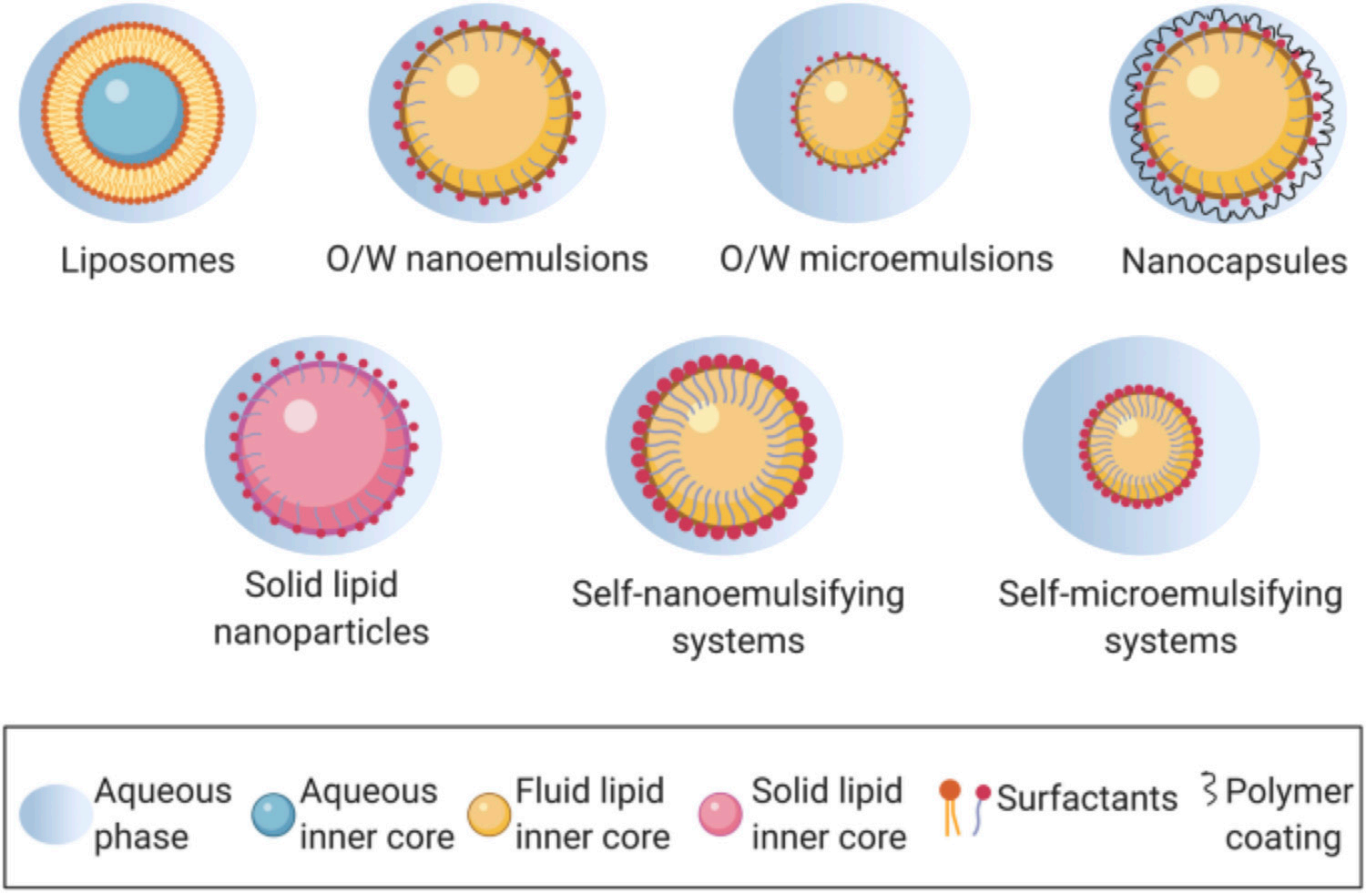
Lipid-based nanocarriers in oral drug delivery. Reproduced and adapted with permission from Plaza-Oliver et al. (2021a).

Because of the outstanding qualities of their lipid components, which include great adaptability, biocompatibility, and low toxicity, these nanocarriers have gained favour in recent years (Feeney et al., 2016). Liposomes, nanostructured lipid carriers (NLCs), solid lipid nanoparticle (Lipospheres), solid lipid nanoparticle (SLM), nano emulsions, and self-nano and micro emulsifying drug delivery systems are all examples of lipid-based nanocarriers (Figure 2).

#### Lipid nano capsule

Lipid nano capsules (LNCs) are a novel type of nanocarrier with a solid outer shell and oily core that has developed to alleviate the drawbacks of the previously stated nanocarriers, such as low loading, limited stability, and the need for organic solvents. LNCs are considered to blend the structural elements of polymeric nanoparticles and liposomes. They consist of an emulsifying agent, an oily core, and an external shell of solid lipids (Anton et al., 2008; Battaglia and Ugazio, 2019). LNCs were very stable and produced without the use of solvents. LNCs showed advantage such tiny particles with a narrow size distribution, elevated drug loading, prolonged release of drug, and an industrially practical manufacturing procedure. Longer plasma circulation was made possible by the polyethylene glycol chains on the surface of LNCs, which cleverly avoided reticuloendothelial absorption. These features all work together to make LNCs sophisticated lipid nanocarriers (Tamjidi et al., 2013; Niu et al., 2016). The biodegradability and biocompatibility of LNCs, along with their physical stability for 1 year and 6 months, are their key advantages (Mouzouvi et al., 2017; Dabholkar et al., 2021). P-gp limits the oral bioavailability of medication. These issues can be solved by creating medications in the form of LNCs (Huynh et al., 2009; El-Moslemany et al., 2016).

The anticancer medication miltefosine is a substitute therapy for Schistosoma mansoni mass chemotherapy in mice. LNCs with a size range of 40–55 nm were available. The overall worm burden of the immature and invasive stages, as well as the quantity and size of granulomas, significantly decreased in mice. Albendazole LNCs exhibit 98% entrapment efficacy, substantial drug loading, and sustained release action when administered orally to mice infected with Echinococcus granuloses (Pensel et al., 2015).

#### ISAsomes as lipid nanocarriers for oral drug delivery

Emulsified microemulsions (EMEs), micellar cubosomes, hexosomes, and Cubosomes are all members of the ISAsomes (Internally Self-Assembled Somes) family unit of structurally tuneable nanoparticles that contain internally inverted micellar phases or non-lamellar liquid crystalline phases (Azmi et al., 2015; Murgia et al., 2020). Inverse discontinuous hexagonal (H2), cubic Fd3m phases, inverse microemulsions (w/o microemulsions, L2 phases), and Inverse bi-continuous cubic (Q2) phases are among the interior structures of these nano-self-assemblies (Yaghmur and Glatter, 2009; Glatter and Salentinig, 2020). These colloidal nano-objects, which are also biologically significant, are created during the digestion of triglyceride-containing foods such milk and mayonnaise and model food emulsions, according to several earlier papers (Hempt et al., 2020). They are said to act as nanostructured carriers for the delivery of vitamins that are not particularly water soluble (Salentinig, 2019). Earlier studies suggested that numerous medications, including, doxorubicin, cinnarizine and 20(S)-protvopanaxadiol added to hexosomes or cubosomes, would have increased bioavailability and prolonged release (Barauskas et al., 2005; Otte et al., 2018; von Halling Laier et al., 2019). Gefitinib Loaded Nanosized Cubosomes (GFT CNPs) were created by El Shenawy et al. using the emulsification technique (Morsi et al., 2014). Which demonstrate the anticancer efficacy of Gefitinib when taken orally utilising lipid nanocarrier cubosomes. Histopathological analysis after GFT-CNPs treatment showed a rise in cancer tissue and noticeably less localised infiltration in the lamina propria. According to this study’s findings, GFTCNPs could be used as an oral vesicular system to treat colon cancer (El-Shenawy et al., 2023).

#### Lipid coated mesoporous silica nanoparticles for oral drug delivery

**FIGURE 3:**
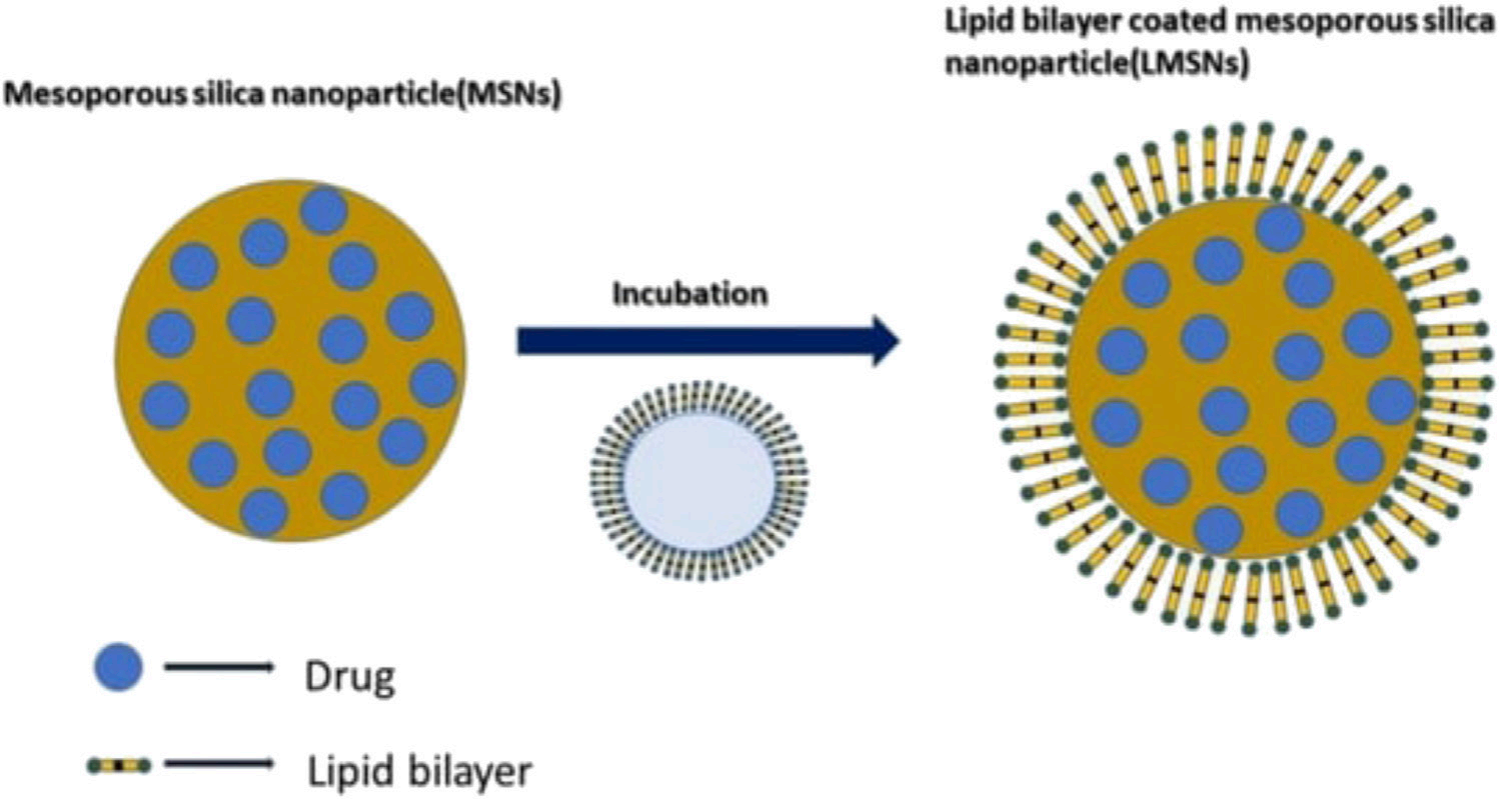
Mechanism of lipid coating in the Mesoporous Silica Nanoparticles. Reproduced with permission from Sreeharsha et al. (2022).

Mesoporous silica nanoparticles (MSNs), also known as silica nanoparticles containing mesopores, have gained popularity recently. Its traits, such as the uniform and adjustable pore size, the ease with which the surface, interior, and exterior pores can be independently functionalized, and the pore release gating mechanism, set it apart as a special and drug carrier (Narayan et al., 2018) (Figure 3). These carriers have been effectively employed by scientists to load a wide range of substances, including medications and macromolecules like protein, DNA, and RNA (Hanafi-Bojd et al., 2016; Möller et al., 2016). Ongoing research is being done to assess the possible new uses for MSNs in medicine delivery. To further understand how MSNs can improve a medication’s solubility and regulated and sustained drug delivery mechanism, numerous studies on MSNs are now being conducted (Maleki et al., 2017; Riikonen et al., 2018). For oral distribution of Vancomycin, John Ndayishimiye et al. created and surface-engineered silica nanoparticles to encapsulate and enhance permeability. The author created SNPs with varying morphologies and pore sizes, altered their surface charges and polarities, and then assessed the effects of these changes on the permeation behaviour and release of loaded Vancomycin. Van-loaded SNPs showed strong loading and a regulated release behaviour in comparison to pure Van. Van-loaded SNPs dramatically improved the permeability of Van across an epithelial cell monolayer, and this improvement was reliant on the pore sizes, morphologies, and surface chemistry of the SNPs. Despite the fact that the Van-loaded SNPs exhibited higher Papp (apparent permeability) values than pure Van and dramatically increased van permeability in the Caco-2 cell model (Duan et al., 2020).

## Methodologies for the formulation of solid and semisolid lipid-based formulations

There are various methodologies available for the formulation of solid and semisolid lipid-based formulations. These methods enable the incorporation of lipid-based carriers into solid or semisolid forms to enhance drug delivery. Additionally, the choice of solidification agents is crucial in achieving the desired formulation characteristics. Here are some common methodologies and solidification agents used in the formulation of solid and semisolid lipid-based formulations.

### High-pressure homogenization or HPH

HPH is a method that pushes a liquid or dispersion through a tiny, micrometer-sized orifice at high pressure (100–2,000 bar) to produce submicron-sized particles. A cavitational forces and strong shear stress break down the particles, results with reduced particle size (Gardouh et al., 2013; Duan et al., 2020). HPH can be performed either at or below room temperature; these conditions are referred to as hot-HPH and cold-HPH, respectively. The drug is disseminated in the melted lipid in the first phase of both techniques by heating the lipid(s) and drug(s) to a temperature that is around 5°C–10°C higher than the melting point of the lipid. In the second step of the HPH process, the aqueous phase containing the amphiphile molecules is combined with the lipid phase (at the same temperature as the lipid melting), and the hot pre emulsion is made using a high-speed stirring apparatus (Sezer, 2014). However, due to the destruction of heat-sensitive pharmaceuticals, this method cannot be used with them. Additionally, increasing the number of rotations or pressure of homogeneity results in an increase in particle size (Svilenov and Tzachev, 2014). However, Van-An Duong et al. produced lipid nanoparticles using cold-HPH to circumvent such restrictions. For improved entrapment effectiveness and sustained-release distribution of ondansetron hydrochloride, cold high-pressure homogenization was used to load lipid carriers into nanostructures (NLCs) (Duong et al., 2019a).

#### O/W micro-emulsion breaking method

The microemulsion was created using this technique by combining a melted lipid with a drug, surface active agents, and co-surface active agents mixture that had been preheated to the lipid’s melting point (Kumar and Randhawa, 2013). The resulting microemulsion was then dispersed in water at temperatures between 2°C and 10°C. The formulations of SLNs made utilising the microemulsion method included stearyl alcohol, cetyl alcohol, trilaurin, stearic acid, and other lipids. There were also added tween 80, tween 20, sodium cholate, sodium deoxycholate, and other surfactants. The end product must be concentrated using ultrafiltration or lyophilization because this method creates an extremely diluted SLNs dispersion (Kumar and Randhawa, 2013). The high concentration of the surface-active agents, and co-surface-active agents in this approach is its main disadvantage.

#### Solvent emulsification–diffusion technique

In this procedure, the lipid is dissolved in a water-saturated organic solvent, and the resultant solution is emulsified with organic solvent and water while being stirred. By adding water to the created emulsion, which encourages the organic phase to permeate into the continuous phase, lipid nanoparticles are produced. With a cut-off of around 100,000 kD, ultrafiltration and a dialysis membrane can be used to purify the SLN dispersion (Trotta et al., 2003). Ibuprofen was encapsulated into solid lipid nanoparticles by Wesley N. Omwoyo et al. using the emulsification solvent evaporation approach to enhance drug absorption and regulated and sustained release of the medication (Omwoyo and Moloto, 2019).

#### Solvent injection method

In this method, the lipids are first solubilised in a water-miscible solvent before being injected by an injection needle into a swirling aqueous solution, either with or without a surfactant. The amount of injected lipid solution, nature of the injected solvent, the viscosity, the diffusion of the injected lipid solvent phase into the aqueous phase, and the concentration of the injected lipids are the parameters of the process for the synthesis of nanoparticles in this technique (Schubert and Müller-Goymann, 2003).

VA Duong et al. were increased sustained release medication activity and decrease ondansetron dose frequency, ondansetron hydrochloride-loaded nanostructured lipid carriers were prepared using the solvent injection method (Duong et al., 2019b).

#### Water/oil/water (w/o/w) double emulsion method

This method is mostly utilised to create lipid formulations that are packed with biological compounds like peptides and insulin and medications that are water soluble. Insulin is dissolved in the inner acidic phase of the w/o/w multiple emulsion and lipids are dissolved in the water-miscible organic phase to create SLNs using the solvent in water emulsion diffusion method. Then, by adding water to the w/o/w emulsion, SLNs are created. As a result, the organic solvent diffuses into the aqueous phase and the SLNs precipitate. The types of the solvent and drug interactions with the solvent and excipients have an impact on this approach (Naguib et al., 2014).

#### Ultrasonication

The idea behind this method is to use sound waves to reduce the particle size. By simultaneously homogenising under high pressure and sonication, this method creates lipid formulations similar to SLNs, which have a size range of 80–800 nm [126,128]. Using high shear homogenization and ultrasonication, Ajiboye, et al. created olanzapine-loaded nanostructured lipid carriers, and *in-vitro* 89% release was accomplished in 24 h in phosphate buffer pH 7.4 (Ajiboye et al., 2021).

#### Super critical fluid technique

When combined with the ultrasonication process, supercritical carbon dioxide dissolves medications that are lipophilic and can be utilised to create lipid formulations. Matthew A. et al. Using a continuous supercritical CO_2_ aided technique, vitamin D3 and curcumin were co-encapsulated in mixed phospholipid nanoliposomes, and the resulting nanoliposomes exhibit good stability under various stress conditions (Chaves et al., 2022).

#### Membrane contractor technique

In this method, a lipid is passed through membrane pores at a temperature more than its melting point. The resulting droplets of melted lipid are then cycled through water outside the pores, where they are further cooled to room temperature (Chen et al., 2006).

#### Electrospray method

A revolutionary method for creating spherically narrowly dispersed SLNs smaller than 1 mm in size is electrodynamic atomization. This technique is used to produce SLNs in powder form (Sridhar and Ramakrishna, 2013). Zein nanoparticles were created by LA de Almeida Campo et al. using the electrospray technique. Zein is a plant protein derived from Zea mays L., which is utilised to help encapsulate bioactive substances with hydrophilic, hydrophobic, and amphiphilic characteristics (de Almeida Campos et al., 2023).

#### Desolvation technique

The medication is continually mixed into an aqueous gelatin solution using this technique. The aforementioned solution is mixed with a desolvating agent to cause desolvation, which is continued until permanent faint turbidity is reached. The cross-linking agent is then added to the aqueous solution, and the mixture is continuously stirred to harden the nanoparticles. A desolvating agent (such salt solution, alcohol, or acetone) is added to the aqueous gelatin solution to dehydrate the gelatin molecules (Pardeshi et al., 2012). Desolvation technique is used by Shamarekh, Khaled et al. to create gelatin nanoparticles (GNPs) that are uniformly tiny, spherical, and of a uniform size. To create GNPs with predictable characteristics, a precise concentration of freeze-dried high molecular weight gelatin (HMWG) was utilised (Pardeshi et al., 2012).

## Solidification technique of lipid-based formulation

Capsule filling with liquid, solid and semisolid lipid formulations.

By using a technique called solidification, the majority of lipid formulations, including SEDDSs, are created as soft or hard gelatin capsules (Nigade et al., 2012). Self-emulsifying formulations that are liquid or semisolid can be easily enclosed in capsules for oral administration. According to Jannin et al., particularly strong medicines can be easily encapsulated, and reasonably more drug loadings of upto 50% w/w are routinely obtained (Friedl et al., 2021). Additionally, the only restrictions on medication loading are the drug solubility and fill weight (Jannin et al., 2008; Gumaste et al., 2013). These liquid products put into capsules and then sealed by micro-spraying, need a substantially shorter 4 step manufacturing process than conventional semisolid formulations (Patel et al., 2018). S-SEDDS are self-emulsifying components that solidify as liquid or semisolid when applied to powders. They are isotropic combinations of oil, surfactant, solvent, and co-solvents. Traditional SEDDS were created in a liquid form, which had a number of drawbacks including poor stability and drug loading efficiency, a limited number of dosage form options, and irreversible drug or excipient precipitation. Solid-SEDDS (S-SEDDS) was introduced as an effective technique to overcome these limitations. It combines the benefits of solid dosage forms, such as improved stability, portability, and patient compliance, with a significant increase in bioavailability.

### Drug absorption mechanism from S-SEDDS

Under mild stomach disturbance, S-SEDDS made in the form of tablets or capsules first disintegrate and then self-emulsify. Lipids and different lipophilic excipients function through a variety of methods to impact the bioavailability of lipophilic medicines when taken orally. These SEDDS components interact with the enterocyte-based transport system, activate the drug’s lymphatic transport pathway, and alter the environment and composition of the intestine (Maji et al., 2021).

A macrolide antibiotic called azithromycin is used to treat several bacterial infections. Because to the drug’s relatively high molecular weight, poor solubility, slow rate of dissolution, and insufficient intestinal absorption, it has a low oral bioavailability (37%) that has been well-documented. Reem Abou Assi and others created and improved liquid (L) and solid (S) self-emulsifying drug delivery systems (SEDDs) of azithromycin to address these disadvantages. Following a 5-min period, drug dissolving trials revealed that, compared to 11.27% of pure azithromycin, >90% and 52.22% of the liquid and solid SEDD formulations in DW, respectively, were released. This finding suggests that the created SEDDs may improve the oral delivery of the medication. At refrigerator storage temperatures, the compositions remained stable (Abou Assi et al., 2020).

Paclitaxel (Ptx) solid-self-emulsifying drug delivery system (S-SEDDS) was created using the spray drying approach to increase Ptx’s low bioavailability (BA). According to the research, the prepared Ptx-loaded S-SEDDS may provide a promising option for improving BA and directing drug delivery to the Ptx lymphatic system (Cho et al., 2016).

The semi-solid additives must be heated to at least 20°C above its melting point. The drugs are subsequently mixed into the product by the process of stirring. This molten liquid is then cooled to room temperature. The cooled “pre-emulsion” mixture is then filled into the capsules.

The best candidates for this straightforward manufacturing method are low-dose, extremely powerful medications, but there may be incompatibilities between the capsule shells and excipients (Pujara, 2012). Storage temperature is another important factor in preventing drug precipitation at low temperatures. The main drawbacks include slower manufacturing rates, a smaller manufacturing capacity, and more expensive manufacturing expenses than those of ordinary tablets (Sander and Holm, 2009; Oh et al., 2011). Table 3 provides a summary of the most popular hard and soft gelatin capsule examples with lipid-based compositions.

**TABLE 3:** Marketed lipid-based capsule formulations for oral administration.

Trade/Brand name	Drug	Type of capsule	Uses
Neoral (Novartis)	Cyclosporin A	Soft gelatin capsule	Immunosuppressant
Sandimmune (Novartis)	Cyclosporin A	Soft gelatin capsule	Immunosuppressant
Gengraf (Abbott)	Cyclosporin A	Hard gelatin capsule	Immunosuppressant
Convulex (Pharmacia)	Valproic acid	Soft gelatin capsule	Antineoplastic
Targretin (Ligand)	Bexarotene	Soft gelatin capsule	Antineoplastic
Vesanoid (Roche)	Tretinoin	Soft gelatin capsule	Acute promyelocytic leukemia
Rocaltrol (Roche)	Calcitriol	Soft gelatin capsule	Calcium regulator
Norvir (Abbott)	Ritonavir	Soft gelatin capsule	HIV antiviral
Fortovase (Roche)	Sanquinavir	Soft gelatin capsule	HIV antiviral
Kaletra (Abbott)	Lopinavir and Ritonavir	Soft gelatin capsule	, HIV-1 antiviral

#### Spray congealing

This procedure is also known as spray cooling. By spraying molten lipid into a cooling chamber, where it comes into contact with the cool air and hardens into sphere-shaped solid particles, this technology can solidify liquid lipid. The solid particles are collected at the chamber’s base and can either be put to hard gelatin capsules or crushed into tablets. Ultrasonic atomizers are frequently used in this spray cooling technique to create solid particles. The viscosity of the formulation, the melting point of the excipients, and the temperature of the cooling air are the variables that need to be taken into account (Favaro-Trindade et al., 2021). By using a spray cooling approach, Al Zahab et al. created a lipid-based microparticles (MP) that could maintain the release of Vildagliptin (VG) for use as a once-daily treatment for type 2 diabetes mellitus. These microparticles were created by combining different quantities of carbomer 934 NF with the lipid carriers g Compritol^®^ and gelucire^®^50/13 (Al Zahabi et al., 2021).

#### Spray drying

Similar to spray congealing, this process involves varying the temperature of the air inside the atomizing chamber. In this method, the drug solution (drug in water or an organic solvent) is sprayed into a heated air chamber, where the water or organic solvent evaporates and creates solid drug microparticles. In addition to lipid excipients, solid carriers like silicon dioxide can be employed during this procedure. By forming hydrogen bonds with the active ingredient, Gelucire (a lipid excipient) increases drug release and creates stable amorphous drug solids in microparticles form (Chauhan et al., 2005; Porter et al., 2008). For effective encapsulation, process variables such as temperature, pressure, feed rate, airflow rates, and wall material selection are essential (Kandasamy and Naveen, 2022). By using spray drying and freeze-drying techniques appropriate for oral delivery, Yigong Guo, Alberto Baldelli, et al. created high-loading insulin nanoparticles with or without mannitol as cryoprotectants. The results show the fastest release and highest cellular uptake efficacy of insulin with the benefit of greater loading capacity with low excipients requirements and operating costs as compared to conventional freeze drying methods (Guo et al., 2022).

##### Adsorption onto solid carrier

A liquid-lipid formulation is adsorbed using this straightforward and affordable method onto a solid support like silicon dioxide, calcium silicate, or magnesium alumino-metasilicate. The liquid-lipid mixture is added to the carrier after being blended in a blender. The carrier must be chosen to have better post-adsorption flow characteristics and a greater capacity to adsorb the liquid formulation. Cross-linked polymers, inorganic colloidal solids, microporous materials, or nanoparticle adsorbents are some examples of the solid carriers (Sanghai et al., 2013; Guo et al., 2022). Examples comprise.

• Silica, silicates, amorphous or micronized silica (Sylysia^®^) with varying degrees of pore volume, or silicon dioxide (Aerosil^®^), which is fumed silica with different grades of particular surface properties.
• Calcium silicate
• Magnesium trisilicate, magnesium hydroxide, or magnesium aluminometasilicate (Neusilin^®^), may also show various particle sizes and surface characteristics (alkaline or neutral).
• Porous dibasic calcium phosphate anhydrous (Fujicalin^®^)
• Talcum crospovidone, cross-linked sodium carboxymethyl cellulose, and/or cross-linked polymethyl methacrylate

To increase clopidogrel’s bioavailability, Abd-Elhakeem, EM, et al. constructs solid self-nano emulsifying drug delivery systems (S-SNEDDS). Clopidogrel S-SNEDDS must be prepared by being adsorbed onto solid Aeroperl^®^ 300. SNEDDS-8 (SII8), which comprised 10% Capryol TM 90, 10% Cremophore EL, and 80% Transcutol HP, had the lowest globule size and the maximum percentage of drug release, according to the data (Abd-Elhakeem et al., 2019).

##### Wet or melt granulation

Melt extrusion, often referred to as extrusion-spheronization, is one of the procedures being researched for the production of self-emulsifying pellets. Extrusion is a solvent-free method for creating an aggregate from raw material with plastic qualities that has cylindrically formed granules of consistent density. The extrudate from the lipid-based formulations is broken down and converted into spherical particles through continual agglomeration and spheronization after being combined with a carrier or adsorbent excipient. These spheroids or pellets frequently have a restricted range of particle sizes, great flowability, and low friability. Additionally, it is asserted that the approach makes high drug loading easier (Patel et al., 2008; Mandić et al., 2017).

The advantages of the melt extrusion process include minimal equipment needs, superior mixing results, and reduced cross-contamination because of its closed design. The parameters are easy to control, and the processing time is brief. A high energy input is needed, heat-sensitive materials cannot be used, only a small number of polymers can be used, and many ingredient properties, such as stability, flow properties, wettability, chemical and physical interactions, etc., must first be thoroughly studied (Tan et al., 2013; Kovačević et al., 2022). In contrast, melt extrusion has some drawbacks. To enhance the solubility of inadequately water-soluble carvedilol, Kovacevic M. et al. developed self-micro emulsifying drug delivery systems (SMEDDS) granules using wet granulation techniques. Mesoporous carriers are utilised for the solidification of SMEDDS (Kovačević et al., 2022).

#### Conversion technique and use of cryo-preservation for converting liquid lipid to solid

To increase the physicochemical stabilities of LBDDS and boost commercialization, numerous efforts have been made to transform liquid lipid systems into solid oral dosage forms. The conversion methods include rotary evaporation, lyophilization, and spray drying.

#### Spray drying

It is the technique that is most frequently employed to transform liquid lipids into solid oral dose forms. Compared to the other conversion process, spray drying is a convenient system. Using this method, liquid lipid compositions are converted into freely flowing powder or granules. On the other hand, the process has several downsides, such as a reduction in Yield production (50%–70%) and being inappropriate for items that are susceptible to heat and oxygen (Ding et al., 2022).

Amorphous and crystalline lutein-loaded microencapsulated powder was created by Ding, Zhuang, and others using the spray drying process to increase bioavailability, water solubility, and chemical stability. In contrast to the crystalline formulation, amorphous lutein nanoparticles demonstrated strong breakability (478.8 nm within 20 min) and quicker dissolving rates. During stability testing, the amorphous formulation demonstrated slower rates of degradation, with decay constants k of 0.03 and 0.07 at 25°C and 40°C, respectively (Degobert and Aydin, 2021).

##### Lyophilization

Freeze-drying, sometimes referred to as lyophilization, is the method of choice for creating pharmaceutical lipid formulations that are unstable in an aqueous media and thermosensitive. In the dried state, this guarantees stability and extended storage. The solvent is first frozen in the lyophilization process, and then it is sublimated away under vacuum. The process of freeze drying typically involves three steps: freezing (solidification), primary drying (ice sublimation), and secondary drying (desorption of unfrozen water). Lyophilization is an alternative to spray drying for items that need to be oxygen sensitive. The approach has drawbacks such a lengthy processing time, a complex process, and high maintenance costs (Ding et al., 2022). To increase the oral bioavailability of zotepine, Nagaraj, et al. created Zotepine loaded lipid nanoparticles (ZT-SLN) for oral administration. By freezing the optimised ZT-SLN formulation at or below 80°C for an extended period of time and lyophilizing it under applied vacuum, the author lyophilized the formulation (Nagaraj et al., 2020).

#### Characterization of lipid-based and lyophilized vesicular drug delivery system

Characterization of lipid-based and lyophilized vesicular drug delivery systems involves assessing various parameters to understand their physical properties, stability, drug encapsulation efficiency, release kinetics, and other relevant attributes. The vesicular drug delivery system involves liposomes, niosomes, transferosomes, pharmacosomes, colloidosomes, herbosomes, sphinosomes. Here are some common characterization techniques for lipid-based and lyophilized vesicular drug delivery systems.

##### Appearance

Examining the appearance for homogeneity and colour at equilibrium can be done using a calibrated glass cylinder or clear glass container (Wei et al., 2005).

##### Colour, odour, and taste

These characteristics are particularly crucial for formulations of oral lipids. Variations in particle size, crystal habit, and subsequent particle disintegration are typically the causes of variations in taste, particularly for active substances. Chemical instability can also be a factor in flavour, odour, and colour variations (Prabhu et al., 2005).

##### Self-dispersion and particle size

Analysing the dispersion rate and consequent particle size is crucial for lipid-based systems. The ideal analysis could determine the nanoparticle size and provide information on the particle’s concentration, shape, and surface properties. Since mean droplet size has a substantial impact on drug release and the lipolysis process, it can be used to assess the effectiveness of lipid formulations like sS-SEDDS. Methods like laser diffraction and Coulter counting photon correlation spectroscopy are widely employed to measure the mean droplet size for diluted SEDDS. These days, this can be accomplished using technological techniques like size exclusion chromatography (SEC), which has a variety of analytical capabilities (Fan et al., 2021).

##### Density

A key component of the formulation is its density or specific gravity. High-accuracy hydrometers can be used to measure density at a given temperature, and a decline in density is often an indication that there is trapped air inside the formulation’s structure (Pujara, 2012).

##### pH value

The pH value of an aqueous formulation should be determined using a pH metre at a certain temperature after settling equilibrium has been reached in order to reduce “pH drift” and electrode surface coating with suspended particles. Neutral electrolytes should not be added to the formulation’s external phase to stabilise the pH because they alter the suspension’s physical stability (Fan et al., 2021).

#### Viscosity measurements

A Brookfield viscometer, a rheometer comprising a cone, plate, and revolving spindle, is used to measure the viscosity of diluted lipid formulations that are microemulsions. While very viscous lipid formulations like SNEDDSs are challenging to encapsulate due to flowability challenges, low-viscosity formulations raise concerns regarding capsule leakage (Buya et al., 2020).

#### Droplet size, zeta potential, and poly dispersibility index

The surface charge of the produced droplets in the dispersion medium is determined by their zeta potential, which also shows how stable they are. It is determined by keeping track of the droplets’ electrophoretic motion. The charge on the droplet is significant for improving drug absorption even though in the instance of SEDDS, zeta potential is not more important for assessing the stability of the emulsion. The interaction between positively charged droplets and the negatively charged membrane is what causes this (Maji et al., 2021). Dynamic light scattering, a Zeta Potential analyzer, was used to calculate the droplet size, zeta potential, and polydispersibility index (PDI) of the lipid formulations (Mahmood et al., 2022).

#### *In vitro* dissolution studies

Lipid-based medication delivery systems can be assessed *in vitro* using lipid digestion models. In order to assess an excipient’s performance during formulation creation and to predict *in vivo* performance, it is crucial to design an *in vitro* dissolution testing procedure. This procedure is referred to as “simulated lipolysis release testing.” The goal of this system’s operation is to keep the pH constant throughout any reaction that produces or consumes hydrogen ions. When a deviation is found, it is corrected for by adding reagent. The model is created from a tube that has been temperature-controlled to 37 ± 1^∘^C. The model consists of a temperature-controlled vessel (37 ± 1^∘^C) containing a simulated intestinal fluid made of bile salt (BS), phospholipids (PL), and digestion buffer. In order to begin the digestion process, pancreatic lipase and colipase were given to this model along with a fluid lipid-based formulation. Fatty acids are released as soon as digestion gets going, which briefly lowers pH. An equimolar amount of sodium hydroxide is introduced to titrate the produced fatty acids by the auto burette in order to prevent a change in the digesting medium’s pH from a predetermined pH value. By monitoring the rate of sodium hydroxide addition and taking into account the stoichiometric connection between fatty acids and sodium hydroxide, the degree of digestion can be determined (Prabhu et al., 2005).

#### *In vivo* studies

By carrying out the necessary *in vivo* studies, the impact of excipients on drug bioavailability and pharmacokinetic profile can be assessed. A thorough investigation of intestinal lymphatic absorption is necessary because lipid-based formulations increase medication bioavailability by boosting intestinal uptake. Lack of clinical data, differences in technique, and the use of different animal models have made it difficult to conduct studies on drug transport by the lymphatic system. Lipid-based formulations in capsules are commonly used to improve the delivery of poorly water-soluble drugs. These formulations, also known as lipid-based drug delivery systems (LBDDS), use lipids as carriers to enhance the solubility, absorption, and bioavailability of such drugs. Lipids are a class of compounds that are soluble in organic solvents and insoluble in water (Huang et al., 2021).

Here are some key points regarding the use of lipid-based formulations in capsules for improving the delivery of poorly water-soluble drugs:

*Solubilization*: Lipids can solubilize poorly water-soluble drugs by incorporating them into lipid matrices or forming drug-lipid complexes. This enhances drug solubility and dispersibility, making it easier for the drug to dissolve in the gastrointestinal (GI) fluids upon administration (Nanjwade et al., 2011; Kalepu et al., 2013b).

*Enhanced absorption*: Lipid-based formulations can promote the absorption of poorly water-soluble drugs by facilitating their transport across the GI tract. Lipids are natural constituents of the body and are easily assimilated, which allows for better drug uptake. Furthermore, lipids can mimic physiological lipids in the GI tract, promoting the drug’s affinity for absorption pathways (Ameta et al., 2023).

*Lymphatic transport*: Lipid-based formulations can exploit the lymphatic transport pathway, bypassing the liver’s first-pass metabolism. This route can be advantageous for drugs that undergo extensive metabolism or have poor oral bioavailability. Lipids can form lipoproteins that are taken up by the lymphatic system, allowing drugs to directly enter the systemic circulation (Nakmode et al., 2022a).

*Sustained release*: Lipid-based formulations can be designed to provide sustained release of drugs, ensuring prolonged therapeutic levels. By incorporating drugs into lipid matrices or liposomes, the release of drugs can be controlled, providing a prolonged and controlled drug delivery profile.

*Protection and stability*: Lipids can act as protective barriers for poorly water-soluble drugs, shielding them from harsh GI conditions and enzymatic degradation. This protection can enhance drug stability, preserving its integrity until it reaches the site of absorption (Alqahtani et al., 2021).

*Formulation flexibility*: Lipid-based formulations offer flexibility in terms of formulation design. They can be formulated as self-emulsifying systems, solid lipid nanoparticles, nanostructured lipid carriers, or liposomes, depending on the specific drug and desired delivery requirements.

It is important to note that the selection of appropriate lipids, excipients, and formulation strategies should be based on the physicochemical properties of the drug and the desired drug delivery outcomes. Lipid-based formulations have shown great potential in improving the delivery of poorly water-soluble drugs and are widely used in pharmaceutical research and development. The clinical trials based on some liposomal drugs used in treatment of cancer and fungal infections are listed in Table 4.

**TABLE 4:** Clinical trials of lipid-based formulation.

Clinical trial	Drug/Target	Phase	Indication	Status	Ref.
NCT00024492	Mitoxantrone	Phase 1	Tumors	Completed	Lamichhane et al. (2018)
NCT00080418	Paclitaxel	Phase 1	Metastatic breast cancer	Completed	Bulbake et al. (2017)
NCT00004083	Cisplatin	Phase 2	Ovarian cancer	Completed	Lamichhane et al. (2018)
NCT02181075	Doxorubicin	Phase 1	Liver tumours	Completed	Middleton (2023)
NCT01259713	Amphotericin B	Phase 3	Fungal infections	Completed	Hawkins (2014)
NCT00418951	Voriconazole and amphotericin B	Phase 2	Fungal infections	Completed	Mattiuzzi (2006)

### Challenges of lipid-based drug delivery system

While lipid-based drug delivery systems (LBDDS) have several advantages for improving the delivery of poorly water-soluble drugs, they also present some challenges. Here are some common challenges associated with the formulation of lipid-based drug delivery systems in capsules:

*Formulation complexity*: Developing lipid-based formulations can be intricate due to the wide range of lipids, surfactants, and co-solvents available. Selecting the appropriate components and optimizing their ratios to achieve desired drug solubility, stability, and release characteristics can be a demanding task.

*Manufacturing considerations*: The manufacturing processes for lipid-based formulations may require specialized equipment and techniques. For instance, the preparation of liposomes or solid lipid nanoparticles may involve high-pressure homogenization or sonication. Scaling up these processes can be challenging and costly.

*Physical stability*: Lipid-based formulations can be vulnerable to physical instability, such as phase separation, particle aggregation, or creaming, which can impact drug performance. Thorough formulation design and optimization are necessary to maintain stability throughout the product’s shelf life.

*Bioavailability variability*: The bioavailability of drugs delivered using lipid-based formulations can exhibit variability among individuals. Factors such as bile secretion, digestion, and transport mechanisms can influence drug absorption and overall bioavailability. Understanding and addressing these variables are crucial for consistent and predictable drug delivery.

*Drug loading and payload limitations*: Some lipid-based systems may have limitations in terms of drug loading capacity. The amount of drug that can be incorporated into the lipid matrix or liposomes may be restricted, posing challenges in achieving high drug concentrations in the formulation.

*Drug compatibility*: Certain drugs may demonstrate poor compatibility with lipids or may interact with lipid excipients, leading to drug instability, degradation, or alteration of therapeutic properties. Compatibility studies are necessary to identify potential drug-lipid interactions and mitigate any negative effects.

*Regulatory considerations*: The development and approval of lipid-based formulations may entail additional regulatory considerations compared to conventional dosage forms. The characterization of lipid excipients, stability studies, and compatibility assessments are important aspects that need to be addressed to meet regulatory requirements.

Despite these challenges, lipid-based formulations continue to be an attractive approach for enhancing the delivery of poorly water-soluble drugs. Ongoing research and advancements in formulation techniques aim to overcome these challenges and optimize the performance of lipid-based drug delivery systems (Cattaneo et al., 2010).

## Conclusion and future prospective

In conclusion, lipid-based formulations in capsules offer promising solutions for improving the delivery of poorly water-soluble drugs. By utilizing lipids as carriers, these formulations enhance drug solubility, absorption, and bioavailability. They can also provide sustained release and protection for the drug, while leveraging the lymphatic transport pathway. Despite the challenges associated with formulation complexity, physical stability, and variability in bioavailability, ongoing research and development efforts are focused on overcoming these obstacles. Looking ahead, the future of lipid-based drug delivery systems holds great potential. Advances in lipid-based formulation design, manufacturing techniques, and understanding of drug-lipid interactions will further optimize the delivery of poorly water-soluble drugs. The development of novel lipid-based carriers and strategies for enhanced drug loading, controlled release, and targeted delivery will continue to drive innovation in this field. Additionally, the integration of lipid-based formulations with other emerging technologies, such as nanotechnology and personalized medicine, may open up new avenues for tailored and efficient drug delivery.

Overall, lipid-based drug delivery systems in capsules offer a versatile and effective platform for addressing the challenges associated with poorly water-soluble drugs. They hold promise for improving therapeutic outcomes, expanding treatment options, and advancing the field of pharmaceutical formulation and delivery. With continued research and development, lipid-based formulations are poised to make significant contributions to the delivery of poorly water-soluble drugs in the future. Studies on human bioavailability and studies on the mechanisms of action of lipid-based formulations should be prioritised in future research.
